# Mesangial cell: A hub in lupus nephritis

**DOI:** 10.3389/fimmu.2022.1063497

**Published:** 2022-12-14

**Authors:** Mengdi Liu, Lei Zhang, Yixin Wang, Weijie Hu, Chunhong Wang, Zhenke Wen

**Affiliations:** ^1^ Jiangsu Key Laboratory of Infection and Immunity, Institutes of Biology and Medical Sciences, Soochow University, Suzhou, China; ^2^ Cyrus Tang Hematology Center, State Key Laboratory of Radiation Medicine and Protection, Soochow University, Suzhou, China

**Keywords:** mesangial cells, lupus nephritis, systemic lupus erythematosus, immune complex, tissue resident immunity

## Abstract

Lupus nephritis (LN) is a severe renal disease caused by the massive deposition of the immune complexes (ICs) in renal tissue, acting as one of the significant organ manifestations of systemic lupus erythematosus (SLE) and a substantial cause of death in clinical patients. As mesangium is one of the primary sites for IC deposition, mesangial cells (MCs) constantly undergo severe damage, resulting in excessive proliferation and increased extracellular matrix (ECM) production. In addition to playing a role in organizational structure, MCs are closely related to *in situ* immunomodulation by phagocytosis, antigen-presenting function, and inflammatory effects, aberrantly participating in the tissue-resident immune responses and leading to immune-mediated renal lesions. Notably, such renal-resident immune responses drive a second wave of MC damage, accelerating the development of LN. This review summarized the damage mechanisms and the *in situ* immune regulation of MCs in LN, facilitating the current drug research for exploring clinical treatment strategies.

## Introduction

In the 20th century, Key and Zimmermann discovered the mesangium’s unique structure and defined mesangial cells (MCs) as a particular cell type (1). The mesangium comprises mononuclear stellate cells between the glomerular arteries and the glomerular basement membrane (GBM) (2). It plays a vital role in glomerular functions and is a primary site of injury in glomerular disorders (3). MCs are derived from the Foxd1^+^ precursor cells and are estimated to account for nearly one-third of the total number of glomerular cells (1, 4). Evidence demonstrates that a single hematopoietic stem cell can differentiate into glomerular MCs (5). Notably, alpha8 integrin (Itgα8) is strongly and exclusively expressed in MCs (6), promoting cellular adhesion while inhibiting their migration and proliferation (7, 8).

MCs are damaged in renal diseases, directly or indirectly affecting the functions of the kidney when the lesions occur and eventually causing renal failure. Therefore, understanding the biological features of MCs is particularly important for treating renal diseases. In recent years, researchers have paid more attention to MCs and have acquired many impressive findings.

## MCs in glomerular homeostasis

### MCs maintain glomerular filtration

**Figure 1:**
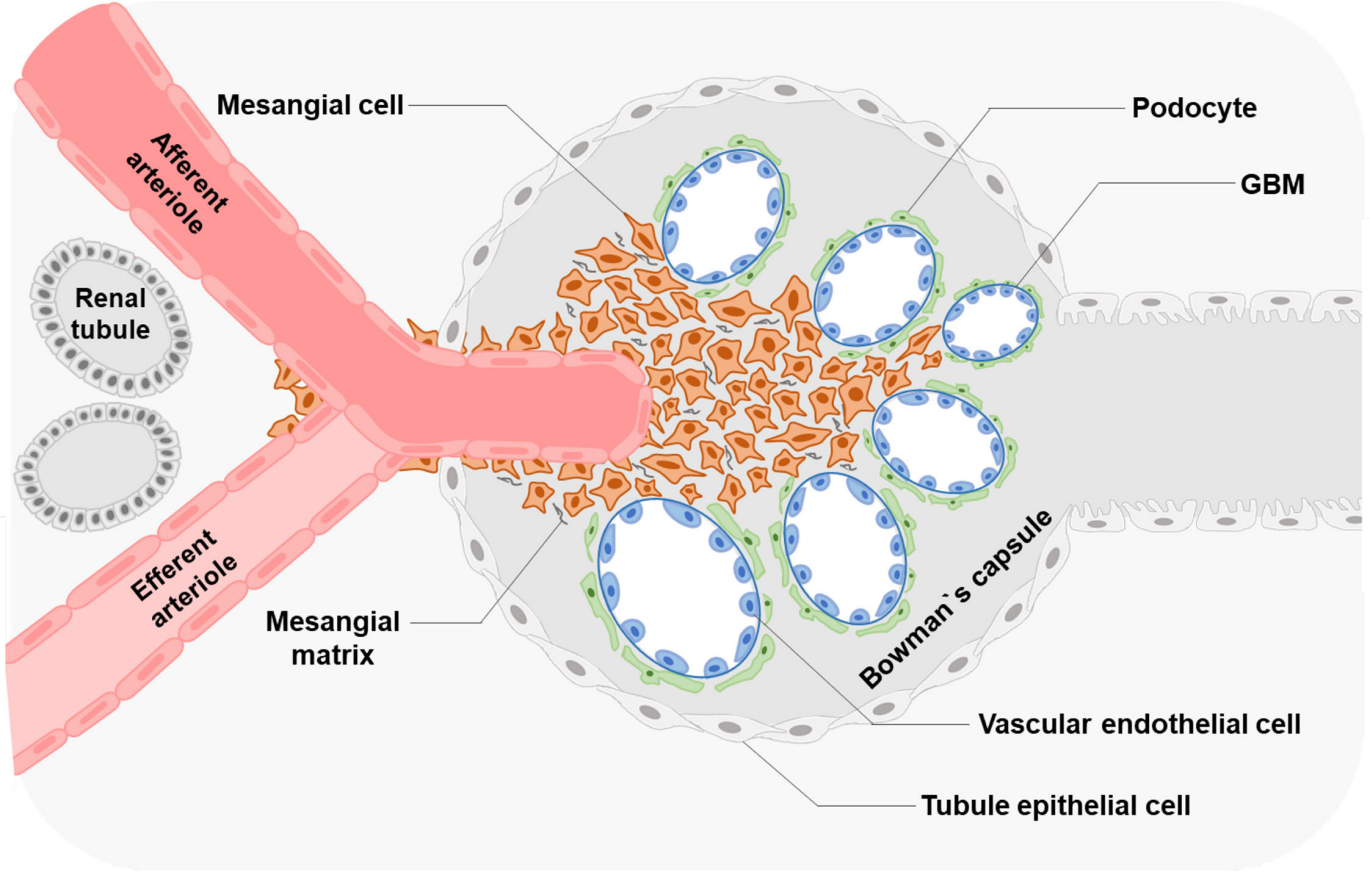
Glomerular structure and MCs' location Glomerular MCs and the mesangial matrix form a stalk that holds and organizes the multiple capillary loops together. MCs extend outside of the glomerulus to the stalk containing the afferent and efferent arterioles.

Morphologically, MCs have a low cytoplasm-to-nucleus ratio and contain fibrils in the cytoplasm (9), supporting the glomerular structure and regulating glomerular filtration (**Figure 1**).

In the glomeruli, endothelial cells are surrounded by GBM and podocytes (9). The regions where no direct contact occurred in the MCs and endothelial walls are called extraglomerular mesangium (10). Extraglomerular mesangium separates MCs and GBM by ECM secreted from MCs (11). These ECM components contain bulk microfibers anchored to the membrane by fibronectin, resulting in a solid structure (12). MCs can exert mechanical traction on the GBM and vascular endothelium through these fiber structures and control capillary pressure and stability, resulting in the appropriate filtration proportion and plasma ultrafiltration rate of the glomeruli (9, 13). Such a connection also provides a corresponding structural basis for the correct screening of macromolecules (14, 15). MCs show structural and functional characteristics of smooth muscle and fibroblasts; thus, they were defined as myofibroblasts (16). Besides, MCs regulate the balance of production and degradation of the mesangial matrix and signaling with other cells to maintain the normal homeostasis of the glomeruli (17–19).

### Roles of MCs in innate immunity

In addition to maintaining glomerular structure and filtration capacity, many studies have demonstrated that MCs can participate in immune responses.

With an evolutionarily conserved defense mechanism, the innate immune system acts mainly by directly disrupting the invading pathogens through phagocytosis and the production of antimicrobial peptides or proteins (20, 21). This relies on recognizing pattern recognition receptors (PRRs) to specific pathogen-associated molecule patterns (PAMPs) (22). MCs express PRRs and participate in innate immune responses. MCs mainly express Toll-like receptors (TLRs) family molecules involved in intrinsic immune regulation. TLRs activate downstream signaling pathways (e.g., interferon regulatory factors (IRF) and nuclear factor kappa-B (NF-κB) pathways) and promote the production of large numbers of adhesion factors, cytokines, and chemokines (CXCLs) (23–25). TLR1-9 mRNA levels are increased in the patients and the MRL/lpr mice as the LN progressed (26). It is shown that certain viral nucleic acids promote LN through nucleic acid-specific TLR (27). MCs express both TLR1-4 and TLR6, especially the highly expressed TLR3 (24, 26). TLR3 signaling contributes to the CXCL1 expression in MCs during the development of inflammatory kidney disease, especially the LN (28). Activation of TLR3 induces MMP-9 in cultured human MCs (HMCs), which could be enhanced by tumor necrosis factor-alpha (TNFα) (29). And, MCs highly express TLR3 to upregulate estrogen receptor alpha (ERα) expression after binding to the ligand, significantly inducing interleukin-6 (IL-6) generation in female LN patients (30). This might explain the predominance of women with lupus. In addition, MCs express NOD-like receptors (NLRs) and are related to NF-κB and transforming growth factor-beta (TGF-β)/Smad signaling pathways (31, 32). MCs also express RIG-I-like receptors (RLRs), which are upregulated upon induction by either interferon (IFN) or double-stranded RNAs (dsRNAs), regulating the generation of cytokines and chemokines (32, 33).

MCs play as non-professional phagocytes. Phagocytosis is not entirely dependent on “professional” phagocytes. There is also a group of “non-professional” phagocytes, such as epithelial cells, endothelial cells, and MCs, exerting phagocytosis function (34, 35). “Non-professional” phagocytes eliminate the deposition of apoptotic cells and matrix components as an essential mechanism for maintaining tissue homeostasis (36, 37). As early as 1962, researchers proposed the presence of phagocytic, resident infiltrating cells in the renal glomeruli (38). Later studies have found that apoptotic MCs were engulfed by neighboring healthy MCs in experimental glomerulonephritis, which contributes to restoring the integrity of glomerular tuft (38). And Itgα8-cytoskeletal interaction facilitated the phagocytosis of MCs (39). After perfusion and enzymatic digestion of the glomeruli, a class of highly adherent phagocytes expressing Fc and C3 receptors was found in the mesangium (40, 41). By injecting the macromolecule dextran, immunoglobulin, and antigen-antibody complexes, it was observed that MCs gradually removed the above substances by electron microscopy (39, 42). MCs perform phagocytosis in both extracellular and intracellular ways. Extracellular decomposition mainly depends on exosomes carrying lysosomal enzymes, and MCs also ingest substances for degradation through endocytosis (40).

MCs are non-professional antigen-presenting cells (APCs). APCs regulate T cells’ activation, differentiation, and proliferation by presenting antigens when renal immune responses occur (43). T-cell activation by APCs requires major histocompatibility complex (MHC) and co-stimulatory molecules (43). The MHC gene complex encodes two major classes of molecules, MHC-I, responsible for the endogenous antigen presentation pathway, and MHC-II, involved in the exogenous antigen presentation pathway (42, 44, 45). Co-stimulatory molecules are surface molecules whose ligands transduce co-stimulatory signals essential for activating T and B lymphocytes (46). Many studies have demonstrated that MCs express both MHC-I and MHC-II molecules, like HLA-DP, HLA-DQ, and HLA-DR, for endogenous or exogenous antigen presentation and cross-presentation (47–49). MCs also express co-stimulatory molecules ICAM-1 and CD80 (B7-1) on the surface (50–52). With the stimulation of IFNγ, the expression of HLA-DP, HLA-DQ, HLA-DR, ICAM-1, and CD80 are markedly enhanced in human MCs, and the activated MCs can process antigen *in vitro*, driving the differentiation of CD4 T cells into Th1 effectors (53). IFNγ-activated mouse MCs also activate CD4 T cell differentiation and proliferation through MHC-II presentation *in vivo* and *in vitro* (52, 53).

## Pathological injuries of MCs in LN

SLE is an autoimmune disease with various clinical manifestations, damaging many systems and organs (54). Loss of immune tolerance to endogenous nuclear material leads to the clinical symptoms (55). The progression of renal disease is the commonly used predictive source of morbidity and mortality in SLE patients (56–58). LN is one of the most severe organ manifestations and presents extensive kidney lesions, which is mainly manifested by the expansion of the glomerular mesangium and accumulation of extracellular matrix secreted from MCs.

### Excessive proliferation of MCs in LN

**Figure 2:**
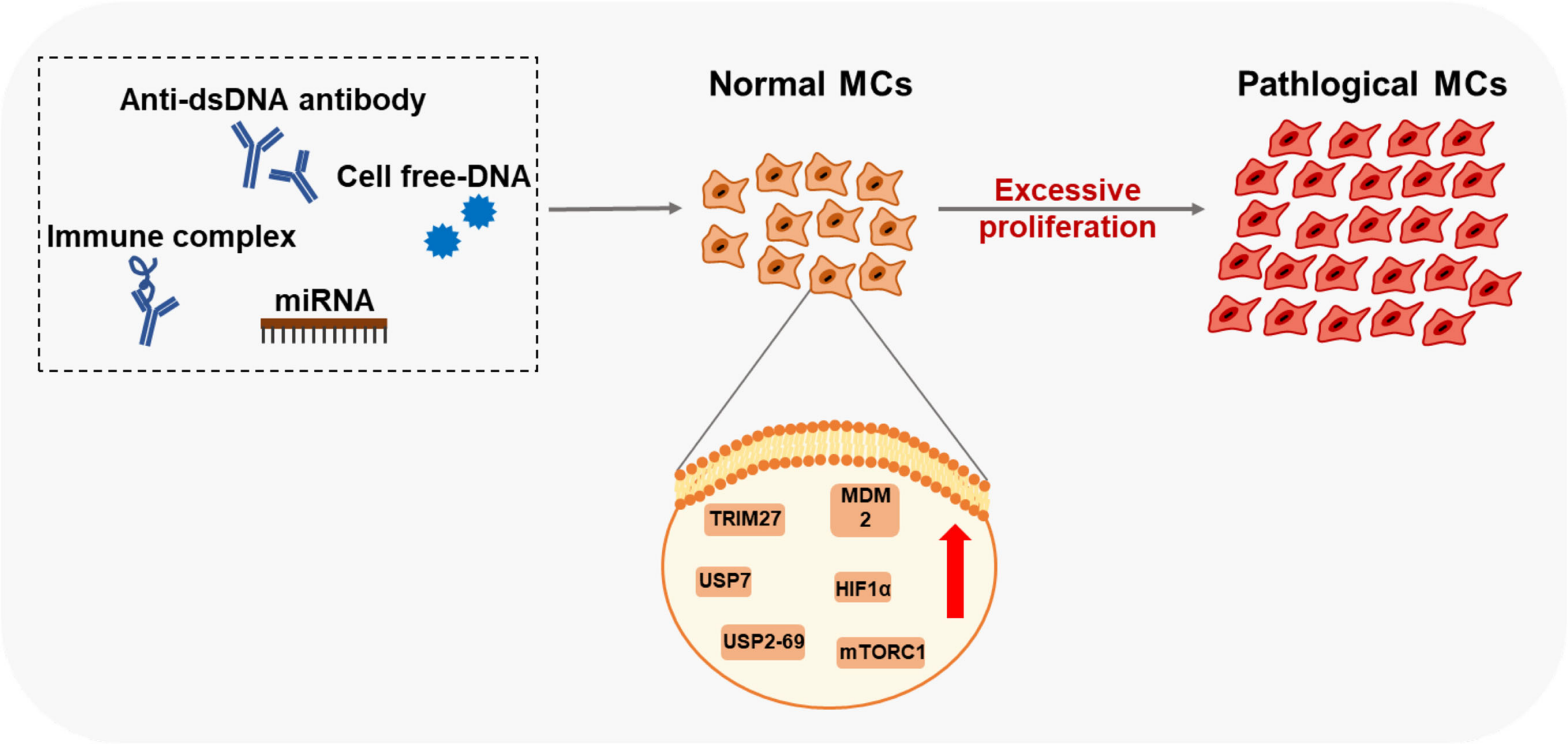
Factors and key proteins that regulate MCs proliferation in LN Anti-dsDNA antibody, cell free-DNA, Immune complex, and miRNA affect the proliferation of MCs through multiple signaling pathways. Many proteins of MCs are aberrantly expressed in LN leading to excessive proliferation.

Excessive proliferation of MCs is one of the representative physiological changes in the progression of LN (59, 60). Self-nucleic acids and IgG anti-dsDNA antibodies can cause the abnormal activation of proliferation-related pathways in MCs (**Figure 2**).

Researchers have found that serum DNA levels correlate with disease activity (61, 62). High values of cell-free DNAs (cDNAs) in patients with SLE were first reported in the 1960s (61). These cDNAs induce MDM2 upregulation, thus negatively modulating both p53 and p21 in human MCs. MDM2 promotes cell cycle change from G0/G1 to the S phase in human MCs (63). However, the mechanisms of how MCs sense DNA or RNA in LN is still unclear, which might involve classic DNA sensors, including TLR9, DAI, AIM2, and the cGAS-STING pathway. Current studies on diabetic nephropathy (DN) have found that TLR9, located in the cell membrane inside, is upregulated in the kidney of experimental mice and the primary mouse MCs treated with high glucose (64). Significant activation of TLR9 is also detected in DN patient-derived MCs, associated with glucose-induced mtDNA damage (65). In the HBV-associated glomerulonephritis (HBV-GN) research, expression of AIM2 was detected by immunohistochemistry in renal biopsies from clinical patients (66). And immunostaining of nephritic kidney sections of autoimmune MRL/lpr mice revealed elevated TLR3 expression in glomerular MCs and recognized poly(I:C) RNA (67).

Anti-double strand DNA (dsDNA) antibodies and pathological IgG are critical pathogenic factors produced by aberrant activated plasma cells in SLE. They could induce the excessive proliferation of MCs. Significant cell proliferation is observed after the soluble IgG treatment in rat MCs (68). Many vital proteins related to organismal regulation are aberrantly expressed in the biopsy specimens from patients with LN. Abnormal activation of mTORC1 causes mesangium expansion, and rapamycin treatment suppresses this effect in Foxd1ER(+) TSC1 mice (69). Tripartite motif-containing 27 (TRIM27), a member of the TRIM protein family, has a strong expression in the LN patients ‘ kidneys, lupus animal models, and human MCs stimulated by LN plasma (60). Downregulation of TRIM27 suppressed the proliferation of mouse MCs in MRL/lpr mice and cultured human MCs by regulating the FoxO1 pathway (60). Hypoxia-inducing factor 1α (HIF1α), highly expressed in LN patients and MRL/lpr mice glomeruli, promotes MC growth under LN progression (70). Deubiquitinases (DUBs) participate in the regulatory networks of plentiful substrates directly implicated in LN progression (71). Ubiquitin-Specific Peptidases7 (USP7) and USP2-69, members of DUBs, are upregulated in LN and related to the proliferation of MCs in MRL/lpr mice and mouse MCs SV40 MES13 cells (71, 72).

IgG anti-dsDNA also drive the expansion of MCs through microRNAs, which are short non-coding RNAs that act as guide molecules in RNA silencing by inducing mRNA degradation or blocking protein translation (73). Research has demonstrated that diverse miRNAs are downregulated in LN induced by anti-dsDNA antibodies and could inhibit MCs’ proliferation through multiple signaling pathways, including miR-10a, miR-16, miR-124, miR-133, miR-155, miR-98-5p, miR-146b-5p, and hsa−miR−371−5p (74–81). Further, some miRNAs exert positive regulatory effects on the proliferation of MCs. As such, miR-148a-3p expression is significantly higher in the glomeruli, and overexpression of miR-148a-3p accelerates MMCs’ expansion (82).

### Increased matrix production of MCs in LN

Another landmark event in the progression of LN is the ECM expansion in the mesangium, derived from an aberrant increasing matrix generation of MCs. Anti-dsDNA antibodies enhance the synthesis of fibronectin (FN), alpha-smooth muscle actin (α-SMA), and TGF-β in cultured human MCs through PKC activation, thus giving rise to the occurrence of kidney fibrosis (83). Oligonucleosomes (ON) are abundant in the circulation and renal biopsies of patients with LN. Rat and mouse MCs stimulated by ON significantly increase the total matrix protein and collagen synthesis (84). The raised expression of TLR2 is observed in glomeruli of LN patients and MRL/lpr mice. TLR2 facilitates glomerular mesangial matrix deposition by activating the MyD88/NF-κB pathway in LN (85).

Excessive proliferation and increased matrix production of MCs are not the only typical features of LN. Still, they are also observed in many other types of nephropathy, such as DN, IgAN, and HBV-GN. High glucose, the most critical risk factor in DN, can drive MCs’ proliferation and secretion of ECM components *via* multiple signal pathways (86–88). IgA immune complexes can induce MC activation and proliferation in IgAN (89, 90). And many viruses have been demonstrated to promote MCs’ proliferation, leading to renal lesions. In addition, factors such as hypoxia also contribute to the proliferation of MCs and the subsequent fibrosis in DN (91, 92). This suggests that different and similar mechanisms exist in various kidney diseases. Although these are pathogenic factors in other conditions, we cannot rule out their possible roles in LN.

## Pathologic MCs shape tissue-resident immunity in LN

### Pathologic MCs produce a mass of inflammatory mediators

Many inflammatory mediators have been implicated in the development and progression of LN, including cytokines, chemokines, and glycosaminoglycans (59). Contents of IFN-α, TNFα, IL-6, and hyaluronan (HA) are increased in serum and renal parenchyma of patients and mice with active LN. Some endogenously inflammatory signaling pathways (e.g., NF−κB, MAPK, JNK, and AKT) induce hyperactivity in renal stromal cells and immune cells by LN pathogenic factors. MCs are one of the essential sources of these inflammatory mediators during the progression of LN (**Table 1**).

**Table 1:** Molecule features of MCs in LN.

ITEM	SLE Patients	Lupus-Prone Mice	Other treatment Related to LN	Other Information
CXCL1	↑ (protein level) (28)		↑ HMCs are treated with poly IC. (protein and mRNA levels) (28)	Regulated by TLR3 signaling (28)
MDM2	↑ (protein level) (63)		↑ HMCs are treated with poly Ic or LN serum. (protein level) (63, 93)	
IFI35	↑ (mRNA and protein levels) (93)		↑ HMCs culture with LN serum. (mRNA and protein levels) (93)	Hypomethylated by MBD2 (93)
IFNγR	↑ (mRNA level) (93)		↑ HMCs culture with LN serum. (mRNA and protein levels) (93)	Hypomethylated by MBD2 (93)
STAT1	↑ (mRNA level) (93)	↑ (MRL/lpr mice, protein level) (93)	↑ HMCs culture with LN serum. (mRNA and protein levels) (93)	Hypomethylated by MBD2. Phosphorylation by IFN-α and IFN-γ (93).
p53			↑ HMCs are treated with poly IC. (protein level) (63)	
p21			↑ HMCs are treated with poly IC. (protein level) (63)	
mTORC1- S6 kinase	↑ (LN-II, protein level) (69)			
TRIM27	↑ (LN-III, IV, protein level) (60)	↑ (MRL/lpr mice, protein level) (60)		Regulated by FoxO1 pathway (60)
HIF1α	↑ (protein level) (70)	↑ (MRL/lpr mice, protein level) (70)		
USP7	↑ (protein level) (71)	↑ (MRL/lpr mice, mRNA and protein levels) (71)		
JMJD3	↑ (protein level) (71)	↑ (MRL/lpr mice, protein level) (71)		USP7 promote the stability of JMJD3 by deubiquitination (71)
p-NF-kB p65		↑ (MRL/lpr mice, protein level) (71)		JMJD3 stabilize NF-kB p65 expression through demethylation (71)
USP2-69	↑ (LN-IV, protein level) (72)		↑ Rat MCs are stimulated with IL-1 and anti-thymocyte serum (ATS). (mRNA and protein levels) (72)	
TLR2	↑ (protein level) (85)	↑ (MRL/lpr mice, protein level) (85)	↑ HMCs culture with LN plasma. (mRNA and protein levels) (85)	
Col IV	↑ (protein level) (85)	↑ (MRL/lpr mice, protein level) (85)		
Annexin II	↑ (protein level) (94, 95)	↑ ( (NZB*NZW)F1/J mice, protein level) (94, 95)		
FN	↑ (protein level) (83)	↑ ( (NZB*NZW)F1/J mice, protein level) (83)	↑ HMCs are stimulated with anti-DNA antibodies. (protein level) (83)	Regulated by PKC (83)
HAS II			↑ HMCs are stimulated with anti-DNA antibodies isolated from LN patients. (mRNA level) (96)	
ER stress pathway			↑ HMCs are stimulated with anti-DNA antibodies isolated from LN patients. (protein level) (97, 98)	
TGF-β	↑ (protein level) (83)	↑ ( (NZB*NZW)F1/J mice, protein level) (83)	↑ HMCs are stimulated with anti-DNA antibodies. (protein level) (83)	Regulated by PKC (83)
IL-1β			↑ HMCs are stimulated with anti-DNA antibodies isolated from LN patients or PDGF-BB. (mRNA and protein levels) (97, 99)	
TNFα			↑ HMCs are stimulated with anti-DNA antibodies isolated from LN patients. (mRNA and protein levels) (97)	
MCP-1			↑ HMCs are stimulated with anti-DNA antibodies isolated from LN patients. (mRNA and protein levels) (97)	
CCL-2			↑ HMCs are stimulated with PDGF-BB (mRNA and protein levels) (99)	
IL-6			↑ HMCs are stimulated with PDGF-BB (mRNA and protein levels) (99)	
APRIL	↑ (LN-III, IV, mRNA and protein levels) (100)			
BLyS	↑ (LN-III, IV, mRNA and protein levels) (100)			
MIF	↑ (mRNA and protein levels) (101)			
NEU1		↑ (MRL/lpr mice and NZM2410 mice, protein level) (102)	↑ Primary MRL/*lpr* lupus-prone MCs are stimulated with HA-IgG (mRNA and protein levels) (102)	
CD40	↑ (markly regulated in LN-III, IV, protein level) (103)			
ICAM-1	↑ (in all LN patients, protein level) (104)			
VCAM-1	↑ (markly regulated in LN-III, M compared to LN- II protein level) (104)	↑ (MRL/lpr mice, protein level) (105)		

Anti-dsDNA antibodies could enhance the inflammation of MCs. They deposit in the glomerular mesangium and bind to MCs surface in both human and murine LN (94, 95). The intracellular inflammatory-related pathways in injured MCs are activated to produce inflammatory mediators induced by pathological anti-dsDNA antibodies (106). Anti-DNA antibodies could also induce hyaluronan synthetase II (HASII) transcription, leading to overexpression of hyaluronan in human MCs (96). Some researchers have reported that anti-dsDNA antibodies isolated from SLE patients caused the activation of the PERK/ER stress pathway, activating NF-κB, a crucial transcription factor in regulating inflammatory processes, leading to the secretion of inflammatory cytokines in HMCs (97, 98). *In vivo* experiments in rats also show that antibodies result in a time- and dose-dependent increase of IL-1β and IL-6 in MCs (97). IL-6, IL-1β, TNFα, MCP-1, and hyaluronan are highly produced in MCs and secreted to the glomeruli. They play crucial roles in the recruitment and retention of lymphocytes at sites of inflammatory renal tissue by binding or inducing chemokines/cytokines synthesis and upregulating the expression of adhesion molecules (96).

Pathological metabolic alterations affect the inflammatory factors production in MCs. Glycosphingolipid (GSL) catabolic pathway is elevated in the kidneys of MRL/lpr mice and human LN patients (102). Neuraminidase (NEU), a key enzyme in the catabolic pathway of GSL, is observed robust activity and expression in LN. High NEU1 activity mediates IL-6 production in the MRL/lpr lupus-prone MCs (102).

In addition to affecting the proliferation and matrix generation, miRNAs also regulate the inflammatory responses of MCs. Many decreased circulating miRNAs might be candidate diagnostic biomarkers for active human LN. MiR-146b, miR-124, and miR-203 attenuate the inflammatory response of MCs by inhibiting the expression levels of TNF receptor-associated factor 6 (TRAF6) in LN (74, 75, 107, 108). MiR-98-5p inhibits the secretion of TNF-α and IL-6 by targeting BACH1 in human MCs (80). Blocking hsa-miR-127-3p could promote the expression of JAK1 and leads to the excessive activation of the IFN-I signaling pathway in LN (109). TLRs activate downstream pathways and stimulate the production of many adhesion factors, cytokines, and chemokines (e.g., TNF-α, IL-12, IL-6) (110). TLR2 upregulation in MCs of LN patients and MRL/lpr mice could also induce inflammatory responses (85).

### Pathologic MCs promote regional immune cell infiltration

MCs could regulate various immune cells *in situ* of LN, but the current understanding of the interactions between MCs and immune cells is still indefinable.

MCs promote the infiltration of macrophages, monocytes, and T cells in the glomerular mesangium through the activation of the NF-κB signaling pathway and the NF-κB-regulated proinflammatory mediators, including IL-6, IL-1β, TNF-α, MCP-1 (111, 112). *De novo* macrophage migration inhibitory factor (MIF) expression is evident in LN MCs examined by *in situ* hybridization and immunohistochemistry staining of biopsies (101). Increased MIF expression is significantly correlated with the reduction of creatinine clearance, immune cells’ accumulation, and the severity of histologic lesions (101). In addition to cytokines, some cell surface proteins of MCs can also induce the infiltration of immune cells through cell-cell contacts. CD40L is a transiently expressed T-cell surface molecule interacting with CD40 on target cells (113). The expression of CD40 is markedly upregulated in MCs with LN and promotes T-cell infiltration in the patients’ glomeruli biopsies (103, 114). The adhesion molecules ICAM-1 and VCAM-1 are upregulated in the MCs of murine models with LN (104). ICAM-1 and VCAM-1 could act as renal adhesion molecules that bind T cells to MHC-II positive cells, thus promoting antigen recognition and renal injury (52, 105).

### Pathologic MCs regulate the differentiation of immune cells in LN

**Figure 3:**
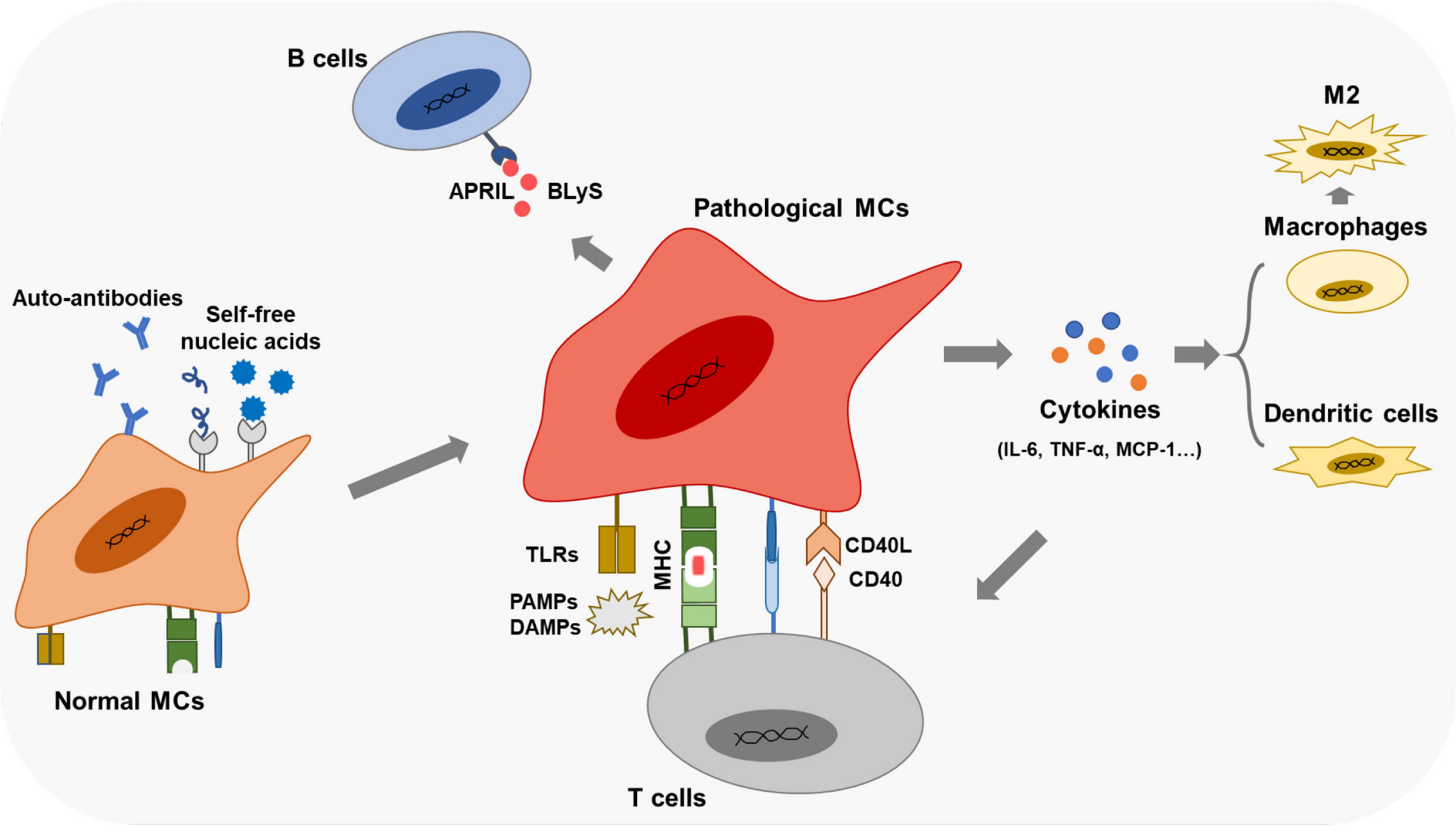
Immune functions of the pathological MCs Pathological MCs with aberrant phagocytic function exert robust expressions of APCs markers (TLRs, MHC, co- stimulators). They secret inflammatory cytokines and chemokines, participate in the renal inflammation effect, and regulate immune cells' infiltration and differentiation by cytokines and cell-cell contacts.

Aside from promoting the infiltration of immune cells in the glomerular mesangium, MCs are also related to the differentiations of immune cells under pathological states (**Figure 3**). Resting human MCs constitutively express IL-6 and chemokine ligand 2 (CCL-2), inducing M2 macrophages polarization under a co-culture system (99). In LN, PDGF-BB-stimulated human MCs rather than resting MCs attenuate classical macrophage activation and drive macrophages into M2 type (99). Human MCs also promote B-cell survival by upregulating APRIL and BLyS, essential for B-cell maturation and activation of plasma cells, facilitating human autoimmune disease progression (100).

## Renal-resident immunity drives a second wave of MC damage in LN

**Figure 4:**
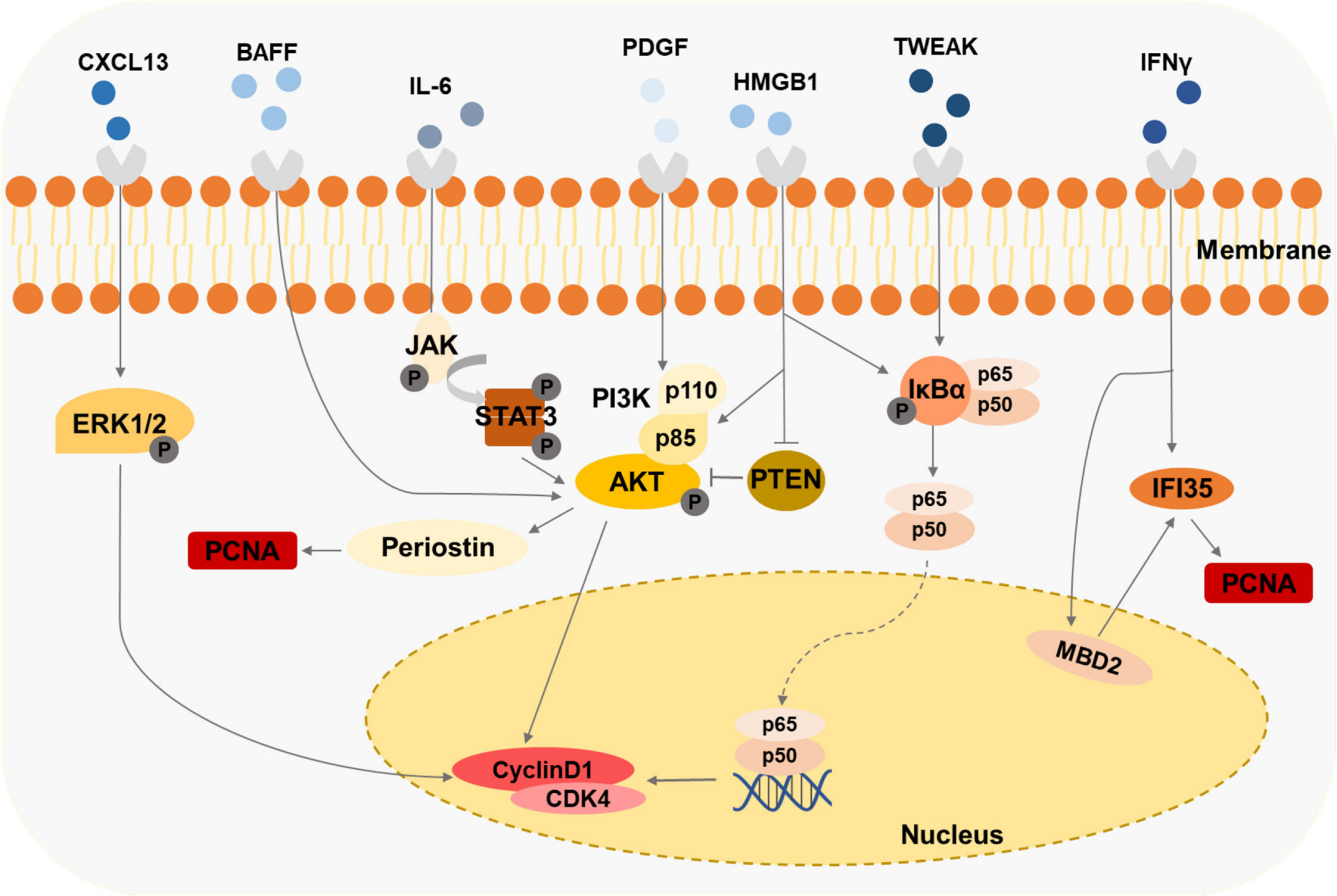
Immune cells-derived cytokines drive the second damage to MCs Cytokines like HMGB1, IFNy, and BAFF induce the proliferation of MCs through various signaling pathways.

MCs regulate *in situ* immunity through paracrine inflammatory mediators or cell-cell contacts. Meanwhile, many cytokines and autoantibodies produced by immune cells further aggravate MCs’ damage in LN (**Figure 4**).

Cytokines, such as PDGF, IL-6, IL-1, and IFN-γ, are accumulated in the kidneys of the mice models and patient biopsies under LN progression (**Table 1**). Several LN-associated cytokines have been shown to contribute to MC proliferation. B cell-activating factor (BAFF), a member of the TNF family, promotes the expansion of human MCs, which is mediated *via* the BAFF-receptor (BAFF-R) (115). CXCL13 accelerates the proliferation of human MCs by triggering extracellular signal-regulated kinase (ERK) tyrosine phosphorylation (116). Contents of IFN-γ and HMGB1 in serum are raised in patients or experimental animal models with LN (93, 117). It has been observed that lots of the IFN-γ/STAT1 pathway-regulated genes are hypomethylated and related to the pathogenesis of LN (118). IFN-induced 35-kDa protein (IFI35) is responsible for these changes in LN (93). IFI35 is regulated by methyl-CpG binding domain protein 2 (MBD2), which could enhance the proliferation of human MCs (93). The researchers have found that HMGB1 expression is specifically increased in lupus patients compared with other renal disease patients (119). HMGB1 promotes the cell cycle transition from G1 to the S phase by the cyclin D1/CDK4/p16 pathway in mouse MCs (117, 120). Furthermore, HMGB1 could mediate mouse MCs’ proliferation through the PTEN/PI3K/Akt/NF-κB signaling pathway and exhibit a synergistic pro-inflammatory effect in MRL/lpr mice (119, 121). Urinary samples of patients with LN and MRL-1pr/1pr mice contain significant IL-6 activity, and the high level of IL-6 is associated with MC proliferation (122, 123). MRL/lpr mice exert enhanced proliferating cell nuclear antigen (PCNA) in MCs mediated by PI3K/Akt/periostin signaling pathway induced with PDGF (124). Tumor necrosis factor-related weak inducer of apoptosis (TWEAK), a member of the TNF-ligand superfamily, is elevated in the blood and urine of patients with LN (125), enhancing NF-κB transcriptional activity and promoting human MC proliferation (125).

## The contribution of MC-related therapeutics to clinical treatment

To achieve rapid remission of active disease, control the progression of chronic kidney disease (CKD), restrain renal flares, alleviate morbidity and mortality, minimize treatment-related toxicity, and preserve fertility, researchers have developed a variety of drugs and therapeutic regimens for LN over the years (126). The present treatment options mainly involve steroids, immunosuppressants, and adjuvant therapies. First-line treatments for LN include steroids, cyclophosphamide (CTX), azathioprine, and mycophenolate mofetil (MMF) (127). Although these medications do not specifically target MCs, inhibition of MCs proliferation is one of their pharmacological effects in controlling the progression of kidney deterioration. The mechanisms of how CTX and MMF inhibit the proliferation of MCs have been validated. CTX arrests the cell cycle in the G1 phase through cell cycle regulators in human MCs (128). MMF is one of the immunosuppressive agents, blocking purine biosynthesis and thereby damaging cell proliferation (129). Besides, recent research has demonstrated that the combination of MMF and tacrolimus (TAC) at the half dose is more therapeutic than monotherapy in inhibiting MC proliferation *in vitro* and *in vivo* (130). TAC is a valid treatment option for SLE patients with renal involvement (131). TAC targets the Smad2 signaling pathway, and MMF targets the p38 signaling pathway, both of which could inhibit MC proliferation (130). The combination of TAC and MMF could significantly benefit patients with LN and shows no severe adverse effects.

Several non-SLE classic drugs developed to attenuate the condition in patients with LN show effective inhibition against MC lesions. The researchers have conducted extensive *in vitro* and *in vivo* studies to verify these medicines’ curative effects and mechanisms. Trifluoperazine (TFP), a calmodulin inhibitor and a classic anxiolytic and antipsychotic drug (132), inhibit human MC proliferation in a dose− and time−dependent manner. By downregulating the Bcl−2 expression and upregulating the Bax expression, TFP promotes cell apoptosis (133). And TFP targets also inhibit the activation of the PI3K/AKT signaling pathway (133, 134). Thus, TFP treatment significantly reduced blood urea nitrogen and serum creatinine levels in lupus mice without apparent side effects (134). Mizoribine (MZR) is a selective inhibitor of the inosine monophosphate dehydrogenase and a key enzyme in the *de novo* pathway of guanine nucleotides (135). MZR downregulates MCP-1 at both mRNA and protein levels in human MCs treated with poly (I:C), which is associated with the pathogenesis of LN (135). ALW (ALWPPNLHAWVP), a peptide with 12 amino acids, inhibits the binding of polyclonal anti-dsDNA antibodies to MCs. ALW attenuates LN lesions, including MC proliferation and inflammatory infiltration in renal tissues of MRL/lpr mice (136). Quercetin is a polyphenol extracted from plants and has many biological activities (137). Quercetin treatment could reduce the expression of pentraxin3 (PTX3) and inhibit the excessive proliferation of human MCs by blocking the NF-κB signaling pathway (138).

## Summary

**Figure 5:**
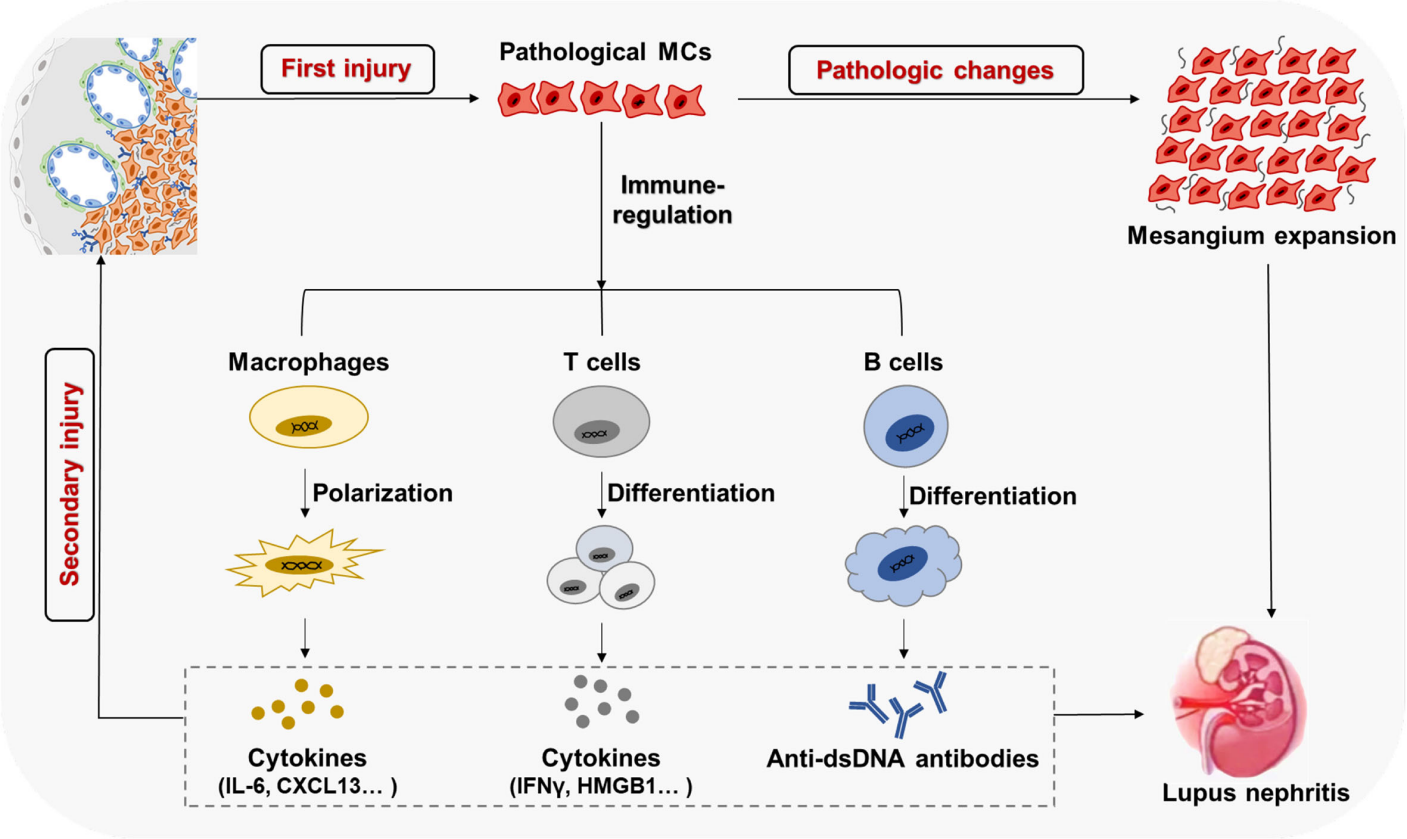
MCs with immune cells form a positive feedback loop to amplify kidney parenchyma in LN ICs' deposition in the mesangium results in the first wave of pathological damage and activation of MCs, causing excessive proliferation and ECM secretion. The activated and pathological MCs participate in local immune regulation, inducing immune cell infiltration and abnormal differentiation. Such renal-resident immune responses increases glomerular local inflammatory mediators and autoantibodies, which drive the second wave of pathological damage to MCs and other stroma cells in the kidney, eventually causing the progress of LN.

MCs are stromal cells that are fatal for renal glomerular homeostasis and the glomerular responses to injury. In LN, the aberrant self-nucleic acids, auto-antibodies, and ICs lead to the first wave of pathogenic damage to MCs. The pathologic MCs proliferate and secrete excess ECM, resulting in kidney dysfunction. In the glomerular microenvironment, MCs not only commit self-injury but also play an essential role in regulating the formation and function of tertiary lymphoid organs in tissues, promoting the abnormal physiological processes of the immune cells by generating massive inflammatory mediators and facilitating immune infiltrations. Such renal-resident immune responses drive the second wave of pathogenic damage to MCs, accelerating kidney dysfunction (**Figure 5**). This might be a universal immunopathological paradigm underpinning the immune-mediated organ damage in human diseases.

Although MCs’ intra-regional immune-related activity has been demonstrated (**Table 1**), precise mechanisms underlying how MCs regulate immune cells in the glomerular region are largely unknown. The immune effects of immune cells activated or amplified by MCs can also affect the morphology and functions of glomerular stromal cells, causing more significant damage to the glomeruli in which MC acts as a critical hub. Precise mechanisms underpinning how MCs maintain homeostasis and how to interfere with pathological MC-immune cell interactions to benefit clinical patients are far less clear. More significant insights into the immunological effects of MCs and their roles in tissue-resident immunity could uncover new treatment strategies to target MCs, revolutionizing the treatment of LN.

## Author contributions

ZW and ML lead the conception and design of the manuscript. ML, LZ, YW, and WH collected and interpreted the relevant literature. ML and LZ drafted the manuscript, while ZW and CW made critical revisions with input from all authors. All authors contributed to the article and approved the submitted version.

## References

[1] Herrera GA. Plasticity of mesangial cells: A basis for understanding pathological alterations. Ultrastructural Pathol (2006) 30(6):471–9. doi: 10.1080/01913120600932594 17182440

[2] Latta H. An approach to the structure and function of the glomerular mesangium. J Am Soc Nephrol (1992) 2(10 Suppl):S65–73. doi: 10.1681/ASN.V210s65 1600139

[3] Zhao JH. Mesangial cells and renal fibrosis. Adv Exp Med Biol (2019) 1165:165–94. doi: 10.1007/978-981-13-8871-2_9 31399966

[4] Boyle SC Liu Z Kopan R. Notch signaling is required for the formation of mesangial cells from a stromal mesenchyme precursor during kidney development. Dev (Cambridge England) (2014) 141(2):346–54. doi: 10.1242/dev.100271 PMC4074211 24353058

[5] Masuya M Drake CJ Fleming PA Reilly CM Zeng H Hill WD. Hematopoietic origin of glomerular mesangial cells. Blood (2003) 101(6):2215–8. doi: 10.1182/blood-2002-04-1076 12433693

[6] Hartner A Schöcklmann H Pröls F Müller U Sterzel RB. Alpha8 integrin in glomerular mesangial cells and in experimental glomerulonephritis. Kidney Int (1999) 56(4):1468–80. doi: 10.1046/j.1523-1755.1999.00662.x 10504498

[7] Bieritz B Spessotto P Colombatti A Jahn A Prols F Hartner A. Role of Alpha8 integrin in mesangial cell adhesion, migration, and proliferation. Kidney Int (2003) 64(1):119–27. doi: 10.1046/j.1523-1755.2003.00057.x 12787402

[8] Marek I Volkert G Hilgers KF Bieritz B Rascher W Reinhardt DP. Fibrillin-1 and Alpha8 integrin are Co-expressed in the glomerulus and interact to convey adhesion of mesangial cells. Cell Adh Migr (2014) 8(4):389–95. doi: 10.4161/cam.28988 PMC4594580 25482639

[9] Makino H. Three-dimensional ultrastructure of rat acellular glomerulus by scanning electron microscopy. J Electron Microsc (Tokyo) (1988) 37(6):294–304. 3244021

[10] Mené P Simonson MS Dunn MJ. Physiology of the mesangial cell. Physiol Rev (1989) 69(4):1347–424. doi: 10.1152/physrev.1989.69.4.1347 2678170

[11] Rupprecht HD Schöcklmann HO Sterzel RB. Cell-matrix interactions in the glomerular mesangium. Kidney Int (1996) 49(6):1575–82. doi: 10.1038/ki.1996.228 8743458

[12] Silva FG Eigenbrodt EH Glass M Taft E. An ultrastructural study of the renal juxtaglomerular apparatus and extraglomerular mesangium in patients with systemic lupus erythematosus. Am J Kidney diseases: Off J Natl Kidney Foundation (1986) 7(1):47–57. doi: 10.1016/s0272-6386(86)80056-7 3942134

[13] Kriz W Elger M Lemley K Sakai T. Structure of the glomerular mesangium: A biomechanical interpretation. Kidney Int Supplement (1990) 30:S2–9. 2259073

[14] Keane WF Raij L. Determinants of glomerular mesangial localization of immune complexes. Role Endothelial Fenestrae Lab Invest (1981) 45(4):366–71. 7300249

[15] Latta H Maunsbach AB. Relations of the centrolobular region of the glomerulus to the juxtaglomerular apparatus. J Ultrastruct Res (1962) 6:562–78. doi: 10.1016/s0022-5320(62)80010-0 14462705

[16] Chung JJ Goldstein L Chen YJ Lee J Webster JD Roose-Girma M. Single-cell transcriptome profiling of the kidney glomerulus identifies key cell types and reactions to injury. J Am Soc Nephrol (2020) 31(10):2341–54. doi: 10.1681/asn.2020020220 PMC7609001 32651223

[17] Baricos WH Cortez SL Deboisblanc M Xin S. Transforming growth factor-beta is a potent inhibitor of extracellular matrix degradation by cultured human mesangial cells. J Am Soc Nephrol (1999) 10(4):790–5. doi: 10.1681/asn.V104790 10203363

[18] Marx M Daniel TO Kashgarian M Madri JA. Spatial organization of the extracellular matrix modulates the expression of pdgf-receptor subunits in mesangial cells. Kidney Int (1993) 43(5):1027–41. doi: 10.1038/ki.1993.145 8510381

[19] Alique M Calleros L Luengo A Griera M Iñiguez M Punzón C. Changes in extracellular matrix composition regulate cyclooxygenase-2 expression in human mesangial cells. Am J Physiol Cell Physiol (2011) 300(4):C907–18. doi: 10.1152/ajpcell.00176.2010 21209362

[20] Bauernfeind F Ablasser A Bartok E Kim S Schmid-Burgk J Cavlar T. Inflammasomes: Current understanding and open questions. Cell Mol Life sciences: CMLS (2011) 68(5):765–83. doi: 10.1007/s00018-010-0567-4 PMC11114650 21072676

[21] Radek K Gallo R. Antimicrobial peptides: Natural effectors of the innate immune system. Semin Immunopathol (2007) 29(1):27–43. doi: 10.1007/s00281-007-0064-5 17621952

[22] Wang L Zhang H Wang M Zhou Z Wang W Liu R. The transcriptomic expression of pattern recognition receptors: Insight into molecular recognition of various invading pathogens in oyster crassostrea gigas. Dev Comp Immunol (2019) 91:1–7. doi: 10.1016/j.dci.2018.09.021 30287242

[23] Robson MG. Toll-like receptors and renal disease. Nephron Exp Nephrol (2009) 113(1):e1–7. doi: 10.1159/000228077 19590236

[24] Tanaka H Imaizumi T. Inflammatory chemokine expression *Via* toll-like receptor 3 signaling in normal human mesangial cells. Clin Dev Immunol (2015) 2013(6):984708. doi: 10.1155/2013/984708 PMC3710592 23935652

[25] Hirono K Imaizumi T Aizawa T Watanabe S Tsugawa K Shiratori T. Endothelial expression of fractalkine (Cx3cl1) is induced by toll-like receptor 3 signaling in cultured human glomerular endothelial cells. Mod Rheumatol (2020) 30(6):1074–81. doi: 10.1080/14397595.2019.1682768 31625434

[26] Patole PS Pawar RD Lech M Zecher D Schmidt H Segerer S. Expression and regulation of toll-like receptors in lupus-like immune complex glomerulonephritis of mrl-Fas(Lpr) mice. Nephrol Dial Transplant (2006) 21(11):3062–73. doi: 10.1093/ndt/gfl336 16954173

[27] Allam R Pawar RD Kulkarni OP Hornung V Hartmann G Segerer S. Viral 5’-triphosphate rna and non-cpg DNA aggravate autoimmunity and lupus nephritis *Via* distinct tlr-independent immune responses. Eur J Immunol (2008) 38(12):3487–98. doi: 10.1002/eji.200838604 19009528

[28] Imaizumi T Aizawa T Segawa C Shimada M Tsuruga K Kawaguchi S. Toll-like receptor 3 signaling contributes to the expression of a neutrophil chemoattractant, Cxcl1 in human mesangial cells. Clin Exp Nephrol (2015) 19(5):761–70. doi: 10.1007/s10157-014-1060-4 25471749

[29] Merkle M Ribeiro A Koppel S Wornle M. Tnfalpha enhances Tlr3-dependent effects on mmp-9 expression in human mesangial cells. Cell Biol Int (2012) 36(12):1155–60. doi: 10.1042/CBI20120282 22950839

[30] Svenson J Cunningham M Dasgupta S Gilkeson GS. Estrogen receptor alpha modulates mesangial cell responses to toll-like receptor ligands. Am J Med Sci (2014) 348(6):492–500. doi: 10.1097/maj.0000000000000339 25343264

[31] Luan P Zhuang J Zou J Li H Shuai P Xu X. Nlrc5 deficiency ameliorates diabetic nephropathy through alleviating inflammation. FASEB J (2018) 32(2):1070–84. doi: 10.1096/fj.201700511RR 29070585

[32] Imaizumi T Tanaka H Tajima A Tsuruga K Oki E Sashinami H. Retinoic acid-inducible gene-I (Rig-I) is induced by ifn-{Gamma} in human mesangial cells in culture: Possible involvement of rig-I in the inflammation in lupus nephritis. Lupus (2010) 19(7):830–6. doi: 10.1177/0961203309360540 20167631

[33] Liu Q Imaizumi T Murakami K Tanaka H Wu Y Yoshizawa T. Dec1 negatively regulates the expression of Cxcl10 and Ccl5 induced by poly ic in normal human mesangial cells. BioMed Res (2017) 38(4):249–55. doi: 10.2220/biomedres.38.249 28794402

[34] Lancaster CE Ho CY Hipolito VEB Botelho RJ Terebiznik MR. Phagocytosis: What’s on the menu? (1). Biochem Cell Biol = Biochim biologie cellulaire (2019) 97(1):21–9. doi: 10.1139/bcb-2018-0008 29791809

[35] Flannagan RS Jaumouillé V Grinstein S. The cell biology of phagocytosis. Annu Rev Pathol (2012) 7:61–98. doi: 10.1146/annurev-pathol-011811-132445 21910624

[36] Kimani SG Geng K Kasikara C Kumar S Sriram G Wu Y. Contribution of defective ps recognition and efferocytosis to chronic inflammation and autoimmunity. Front Immunol (2014) 5:566. doi: 10.3389/fimmu.2014.00566 25426118 PMC4226236

[37] Farquhar MG Palade GE. Functional evidence for the existence of a third cell type in the renal glomerulus: Phagocytosis of filtration residues by a distinctive “Third” cell. J Cell Biol (1962) 13(1):55–87. doi: 10.1083/jcb.13.1.55 19866600 PMC2106064

[38] Baker AJ Mooney A Hughes J Lombardi D Johnson RJ Savill J. Mesangial cell apoptosis: The major mechanism for resolution of glomerular hypercellularity in experimental mesangial proliferative nephritis. J Clin Invest (1994) 94(5):2105–16. doi: 10.1172/jci117565 PMC294654 7962557

[39] Marek I Becker R Fahlbusch FB Menendez-Castro C Rascher W Daniel C. Expression of the Alpha8 integrin chain facilitates phagocytosis by renal mesangial cells. Cell Physiol Biochem (2018) 45(6):2161–73. doi: 10.1159/000488160 29544224

[40] Schreiner GF. The mesangial phagocyte and its regulation of contractile cell biology. J Am Soc Nephrol (1992) 2(10 Suppl):S74–82. doi: 10.1681/ASN.V210s74 1600140

[41] Hinglais N Kazatchkine MD Charron DJ Appay MD Mandet C Paing M. Immunohistochemical study of ia antigen in the normal and diseased human kidney. Kidney Int (1984) 25(3):544–50. doi: 10.1038/ki.1984.52 6204099

[42] Zhao Y Li Q Ouyang Q Wu L Chen X. Activated mesangial cells acquire the function of antigen presentation. Cell Immunol (2021) 361:104279. doi: 10.1016/j.cellimm.2020.104279 33422698

[43] Hamilos DL. Antigen presenting cells. Immunol Res (1989) 8(2):98–117. doi: 10.1007/bf02919073 2659691

[44] Smyth LA Herrera OB Golshayan D Lombardi G Lechler RI. A novel pathway of antigen presentation by dendritic and endothelial cells: Implications for allorecognition and infectious diseases. Transplantation (2006) 82(1 Suppl):S15–8. doi: 10.1097/01.tp.0000231347.06149.ca 16829787

[45] Moreno J Vignali DA Nadimi F Fuchs S Adorini L Hämmerling GJ. Processing of an endogenous protein can generate mhc class ii-restricted T cell determinants distinct from those derived from exogenous antigen. J Immunol (Baltimore Md: 1950) (1991) 147(10):3306–13. 1658143

[46] Vyth-Dreese FA Dellemijn TA Majoor D de Jong D. Localization in situ of the Co-stimulatory molecules B7.1, B7.2, Cd40 and their ligands in normal human lymphoid tissue. Eur J Immunol (1995) 25(11):3023–9. doi: 10.1002/eji.1830251106 7489738

[47] Halloran PF Urmson J Ramassar V Laskin C Autenried P. Increased class I and class ii mhc products and mrna in kidneys of mrl-Lpr/Lpr mice during autoimmune nephritis and inhibition by cyclosporine. J Immunol (Baltimore Md: 1950) (1988) 141(7):2303–12. 3049805

[48] Ikeda M Minota S Kano S. Regulation of mhc class I expression by inflammatory cytokines in rat mesangial cells. Nephron (1997) 76(1):90–5. doi: 10.1159/000190146 9171306

[49] Madrenas J Parfrey NA Halloran PF. Interferon gamma-mediated renal mhc expression in mercuric chloride-induced glomerulonephritis. Kidney Int (1991) 39(2):273–81. doi: 10.1038/ki.1991.33 1825860

[50] Radeke HH Tschernig T Karulin A Schumm G Emancipator SN Resch K. Cd4+ T cells recognizing specific antigen deposited in glomeruli cause glomerulonephritis-like kidney injury. Clin Immunol (Orlando Fla) (2002) 104(2):161–73. doi: 10.1006/clim.2002.5246 12165277

[51] Riser BL Varani J Cortes P Yee J Dame M Sharba AK. Cyclic stretching of mesangial cells up-regulates intercellular adhesion molecule-1 and leukocyte adherence: A possible new mechanism for glomerulosclerosis. Am J Pathol (2001) 158(1):11–7. doi: 10.1016/s0002-9440(10)63938-7 PMC1850278 11141473

[52] Brennan DC Jevnikar AM Takei F Reubin-Kelley VE. Mesangial cell accessory functions: Mediation by intercellular adhesion molecule-1. Kidney Int (1990) 38(6):1039–46. doi: 10.1038/ki.1990.310 1981601

[53] Yu H Cui S Mei Y Li Q Wu L Duan S. Mesangial cells exhibit features of antigen-presenting cells and activate Cd4+ T cell responses. J Immunol Res (2019) 2019:2121849. doi: 10.1155/2019/2121849 31317046 PMC6604415

[54] Modjinou D Gurin L Chhabra A Mikolaenko I Lydon E Smiles S. A case of systemic lupus erythematosus associated with longitudinal extensive transverse myelitis, cerebral neutrophilic vasculitis, and cerebritis. Bull Hosp Joint Dis (2013) 2014) 72(4):294–300. 25986355

[55] Pan XF Gu JQ Shan ZY. Patients with systemic lupus erythematosus have higher prevalence of thyroid autoantibodies: A systematic review and meta-analysis. PloS One (2015) 10(4):e0123291. doi: 10.1371/journal.pone.0123291 25905898 PMC4408090

[56] Cervera R Khamashta MA Font J Sebastiani GD Gil A Lavilla P. Morbidity and mortality in systemic lupus erythematosus during a 10-year period: A comparison of early and late manifestations in a cohort of 1,000 patients. Med (Baltimore) (2003) 82(5):299–308. doi: 10.1097/01.md.0000091181.93122.55 14530779

[57] Croca SC Rodrigues T Isenberg DA. Assessment of a lupus nephritis cohort over a 30-year period. Rheumatol (Oxford England) (2011) 50(8):1424–30. doi: 10.1093/rheumatology/ker101 21415024

[58] Estévez Del Toro M Varela Ceballos I Chico Capote A Kokuina E Sánchez Bruzón Y Casas Figueredo N. Predictive factors for the development of lupus nephritis after diagnosis of systemic lupus erythematosus. Reumatol Clin (2022) 18(9):513–517. doi: 10.1016/j.reumae.2021.08.003 35523640

[59] Anders HJ Saxena R Zhao MH Parodis I Salmon JE Mohan C. Lupus nephritis. Nat Rev Dis Primers (2020) 6(1):7. doi: 10.1038/s41572-019-0141-9 31974366

[60] Liu J Feng X Tian Y Wang K Gao F Yang L. Knockdown of Trim27 expression suppresses the dysfunction of mesangial cells in lupus nephritis by Foxo1 pathway. J Cell Physiol (2019) 234(7):11555–66. doi: 10.1002/jcp.27810 30648253

[61] Hou YQ Liang DY Lou XL Zhang M Zhang ZH Zhang LR. Branched DNA-based alu quantitative assay for cell-free plasma DNA levels in patients with sepsis or systemic inflammatory response syndrome. J Crit Care (2016) 31(1):90–5. doi: 10.1016/j.jcrc.2015.10.013 26589770

[62] Snyder MW Kircher M Hill AJ Daza RM Shendure J. Cell-free DNA comprises an in vivo nucleosome footprint that informs its tissues-of-Origin. Cell (2016) 164(1-2):57–68. doi: 10.1016/j.cell.2015.11.050 26771485 PMC4715266

[63] Zhang CX Chen J Cai L Wu J Wang JY Cao LF. DNA Induction of Mdm2 promotes proliferation of human renal mesangial cells and alters peripheral b cells subsets in pediatric systemic lupus erythematosus. Mol Immunol (2018) 94:166–75. doi: 10.1016/j.molimm.2018.01.003 29324237

[64] Shen J Dai Z Li Y Zhu H Zhao L. Tlr9 regulates Nlrp3 inflammasome activation *Via* the nf-kb signaling pathway in diabetic nephropathy. Diabetol Metab syndrome (2022) 14(1):26. doi: 10.1186/s13098-021-00780-y PMC8815223 35120573

[65] Czajka A Ajaz S Gnudi L Parsade CK Jones P Reid F. Altered mitochondrial function, mitochondrial DNA and reduced metabolic flexibility in patients with diabetic nephropathy. EBioMedicine (2015) 2(6):499–512. doi: 10.1016/j.ebiom.2015.04.002 26288815 PMC4534759

[66] Zhen J Zhang L Pan J Ma S Yu X Li X. Aim2 mediates inflammation-associated renal damage in hepatitis b virus-associated glomerulonephritis by regulating caspase-1, il-1β, and il-18. Mediators Inflammation (2014) 2014:190860. doi: 10.1155/2014/190860 PMC3950499 24701032

[67] Patole PS Gröne HJ Segerer S Ciubar R Belemezova E Henger A. Viral double-stranded rna aggravates lupus nephritis through toll-like receptor 3 on glomerular mesangial cells and antigen-presenting cells. J Am Soc Nephrol (2005) 16(5):1326–38. doi: 10.1681/asn.2004100820 15772251

[68] Zhu LJ Yang X Li XY Liu QH Tang XQ Zhou SF. Suppression of tumor necrosis factor receptor associated factor (Traf)-2 attenuates the proinflammatory and proliferative effect of aggregated igg on rat renal mesangial cells. Cytokine (2010) 49(2):201–8. doi: 10.1016/j.cyto.2009.10.004 19910209

[69] Nagai K Tominaga T Ueda S Shibata E Tamaki M Matsuura M. Mesangial cell mammalian target of rapamycin complex 1 activation results in mesangial expansion. J Am Soc Nephrol (2017) 28(10):2879–85. doi: 10.1681/ASN.2016111196 PMC5619961 28701517

[70] Deng W Ren Y Feng X Yao G Chen W Sun Y. Hypoxia inducible factor-1 alpha promotes mesangial cell proliferation in lupus nephritis. Am J Nephrol (2014) 40(6):507–15. doi: 10.1159/000369564 25531641

[71] Zhang F Zhang B Tang R Jiang H Ji Z Chen Y. The occurrence of lupus nephritis is regulated by Usp7-mediated Jmjd3 stabilization. Immunol Lett (2021) 235:41–50. doi: 10.1016/j.imlet.2021.04.006 33895173

[72] Wang S Wu H Liu Y Sun J Zhao Z Chen Q. Expression of Usp2-69 in mesangial cells in vivo and in vitro. Pathol Int (2010) 60(3):184–92. doi: 10.1111/j.1440-1827.2010.02496.x 20403044

[73] Lee Y Kim M Han J Yeom KH Lee S Baek SH. Microrna genes are transcribed by rna polymerase ii. EMBO J (2004) 23(20):4051–60. doi: 10.1038/sj.emboj.7600385 PMC524334 15372072

[74] Sheng ZX Yao H Cai ZY. The role of mir-146b-5p in Tlr4 pathway of glomerular mesangial cells with lupus nephritis. Eur Rev Med Pharmacol Sci (2018) 22(6):1737–43. doi: 10.26355/eurrev_201803_14589 29630120

[75] Zhang L Zhang X Si F. Microrna-124 represents a novel diagnostic marker in human lupus nephritis and plays an inhibitory effect on the growth and inflammation of renal mesangial cells by targeting Traf6. Int J Clin Exp Pathol (2019) 12(5):1578–88. PMC6947142 31933975

[76] Tangtanatakul P Thammasate B Jacquet A Reantragoon R Pisitkun T Avihingsanon Y. Transcriptomic profiling in human mesangial cells using patient-derived lupus autoantibodies identified mir-10a as a potential regulator of Il8. Sci Rep (2017) 7(1):14517. doi: 10.1038/s41598-017-15160-8 29109423 PMC5673966

[77] Qi H Cao Q Liu Q. Microrna-16 directly binds to Dec2 and inactivates the Tlr4 signaling pathway to inhibit lupus nephritis-induced kidney tissue hyperplasia and mesangial cell proliferation. Int Immunopharmacol (2020) 88:106859. doi: 10.1016/j.intimp.2020.106859 32795896

[78] Huang Z Pang G Huang YG Li C. Mir-133 inhibits proliferation and promotes apoptosis by targeting Lasp1 in lupus nephritis. Exp Mol Pathol (2020) 114:104384. doi: 10.1016/j.yexmp.2020.104384 31987844

[79] Kong J Li L Lu Z Song J Yan J Yang J. Microrna-155 suppresses mesangial cell proliferation and tgf-Beta1 production *Via* inhibiting Cxcr5-erk signaling pathway in lupus nephritis. Inflammation (2019) 42(1):255–63. doi: 10.1007/s10753-018-0889-1 PMC6394596 30209639

[80] Du Y Shi X Li J Jia Y. Microrna-98-5p inhibits human mesangial cell proliferation and tnf-alpha and il-6 secretion by targeting btb and cnc homology 1. Exp Ther Med (2021) 22(6):1436. doi: 10.3892/etm.2021.10871 34721678 PMC8549099

[81] Yao F Sun L Fang W Wang H Yao D Cui R. Hsamir3715p inhibits human mesangial cell proliferation and promotes apoptosis in lupus nephritis by directly targeting hypoxiainducible factor 1alpha. Mol Med Rep (2016) 14(6):5693–8. doi: 10.3892/mmr.2016.5939 27878241

[82] Qingjuan L Xiaojuan F Wei Z Chao W Pengpeng K Hongbo L. Mir-148a-3p overexpression contributes to glomerular cell proliferation by targeting pten in lupus nephritis. Am J Physiol Cell Physiol (2016) 310(6):C470–8. doi: 10.1152/ajpcell.00129.2015 26791485

[83] Yung S Zhang Q Zhang CZ Chan KW Lui SL Chan TM. Anti-DNA antibody induction of protein kinase c phosphorylation and fibronectin synthesis in human and murine lupus and the effect of mycophenolic acid. Arthritis Rheum (2009) 60(7):2071–82. doi: 10.1002/art.24573 19565476

[84] Coritsidis GN Lombardo F Rumore P Kuo SF Izzo R Mir R. Nucleosome effects on mesangial cell matrix and proliferation: A possible role in early lupus nephritis. Exp Nephrol (2002) 10(3):216–26. doi: 10.1159/000058348 12053123

[85] Feng X Yang R Tian Y Miao X Guo H Gao F. Hmgb1 protein promotes glomerular mesangial matrix deposition *Via* Tlr2 in lupus nephritis. J Cell Physiol (2020) 235(6):5111–9. doi: 10.1002/jcp.29379 31667864

[86] Wu Y Zhao Y Yang HZ Wang YJ Chen Y. Hmgb1 regulates ferroptosis through Nrf2 pathway in mesangial cells in response to high glucose. Biosci Rep (2021) 41(2):BSR20202924. doi: 10.1042/bsr20202924 33565572 PMC7897919

[87] Zhang B Shi YQ Zou JJ Chen XF Tang W Ye F. High glucose stimulates cell proliferation and collagen iv production in rat mesangial cells through inhibiting ampk-K(Atp) signaling. Int Urol Nephrol (2017) 49(11):2079–86. doi: 10.1007/s11255-017-1654-3 28748494

[88] Chen Z Gao H Wang L Ma X Tian L Zhao W. Farrerol alleviates high glucose-induced renal mesangial cell injury through the Ros/Nox4/Erk1/2 pathway. Chemico-biol Interact (2020) 316:108921. doi: 10.1016/j.cbi.2019.108921 31838053

[89] Kim MJ McDaid JP McAdoo SP Barratt J Molyneux K Masuda ES. Spleen tyrosine kinase is important in the production of proinflammatory cytokines and cell proliferation in human mesangial cells following stimulation with Iga1 isolated from iga nephropathy patients. J Immunol (Baltimore Md: 1950) (2012) 189(7):3751–8. doi: 10.4049/jimmunol.1102603 22956578

[90] Liang Y Zhang J Zhou Y Xing G Zhao G Liu Z. Proliferation and cytokine production of human mesangial cells stimulated by secretory iga isolated from patients with iga nephropathy. Cell Physiol Biochem (2015) 36(5):1793–808. doi: 10.1159/000430151 26184511

[91] Sodhi CP Phadke SA Batlle D Sahai A. Hypoxia and high glucose cause exaggerated mesangial cell growth and collagen synthesis: Role of osteopontin. Am J Physiol Renal Physiol (2001) 280(4):F667–74. doi: 10.1152/ajprenal.2001.280.4.F667 11249858

[92] Li A Peng R Sun Y Liu H Peng H Zhang Z. Lincrna 1700020i14rik alleviates cell proliferation and fibrosis in diabetic nephropathy *Via* mir-34a-5p/Sirt1/Hif-1α signaling. Cell Death Dis (2018) 9(5):461. doi: 10.1038/s41419-018-0527-8 29700282 PMC5919933

[93] Zhang L Zhu H Li Y Dai X Zhou B Li Q. The role of Ifi35 in lupus nephritis and related mechanisms. Mod Rheumatol (2017) 27(6):1010–8. doi: 10.1080/14397595.2016.1270387 28064541

[94] Yung S Cheung KF Zhang Q Chan TM. Anti-dsdna antibodies bind to mesangial annexin ii in lupus nephritis. J Am Soc Nephrol (2010) 21(11):1912–27. doi: 10.1681/asn.2009080805 PMC3014006 20847146

[95] Salles JP Gayral-Taminh M Fauvel J Delobbe I Mignon-Conté M Conté JJ. Sustained effect of angiotensin ii on tyrosine phosphorylation of annexin I in glomerular mesangial cells. J Biol Chem (1993) 268(17):12805–11. doi: 10.1016/S0021-9258(18)31459-5 7685351

[96] Yung S Tsang RC Leung JK Chan TM. Increased mesangial cell hyaluronan expression in lupus nephritis is mediated by anti-DNA antibody-induced il-1beta. Kidney Int (2006) 69(2):272–80. doi: 10.1038/sj.ki.5000042 16408116

[97] Zhang H Zhao C Wang S Huang Y Wang H Zhao J. Anti-dsdna antibodies induce inflammation *Via* endoplasmic reticulum stress in human mesangial cells. J Transl Med (2015) 13:178. doi: 10.1186/s12967-015-0536-7 26040555 PMC4467615

[98] Yang H Cui J Shi J Yang B Wang M Wu D. Endoplasmic reticulum stress participates in inflammation-accelerated, lipid-mediated injury of human glomerular mesangial cells. Nephrol (Carlton) (2017) 22(3):234–42. doi: 10.1111/nep.12748 26890338

[99] Liao WQ Cui SY Ouyang Q Mei Y Cai GY Fu B. Modulation of macrophage polarization by human glomerular mesangial cells in response to the stimuli in renal microenvironment. J Interferon Cytokine Res (2018) 38(12):566–77. doi: 10.1089/jir.2018.0093 30523751

[100] Neusser MA Lindenmeyer MT Edenhofer I Gaiser S Kretzler M Regele H. Intrarenal production of b-cell survival factors in human lupus nephritis. Mod Pathol (2011) 24(1):98–107. doi: 10.1038/modpathol.2010.184 20890272

[101] Lan HY Yang N Nikolic-Paterson DJ Yu XQ Mu W Isbel NM. Expression of macrophage migration inhibitory factor in human glomerulonephritis. Kidney Int (2000) 57(2):499–509. doi: 10.1046/j.1523-1755.2000.00869.x 10652026

[102] Sundararaj K Rodgers JI Marimuthu S Siskind LJ Bruner E Nowling TK. Neuraminidase activity mediates il-6 production by activated lupus-prone mesangial cells. Am J Physiol Renal Physiol (2018) 314(4):F630–42. doi: 10.1152/ajprenal.00421.2017 PMC5966761 29357434

[103] Yellin MJ D’Agati V Parkinson G Han AS Szema A Baum D. Immunohistologic analysis of renal Cd40 and Cd40l expression in lupus nephritis and other glomerulonephritides. Arthritis Rheum (1997) 40(1):124–34. doi: 10.1002/art.1780400117 9008608

[104] Abd-Elkareem MI Al Tamimy HM Khamis OA Abdellatif SS Hussein MR. Increased urinary levels of the leukocyte adhesion molecules icam-1 and vcam-1 in human lupus nephritis with advanced renal histological changes: Preliminary findings. Clin Exp Nephrol (2010) 14(6):548–57. doi: 10.1007/s10157-010-0322-z 20714774

[105] Wuthrich RP. Vascular cell adhesion molecule-1 (Vcam-1) expression in murine lupus nephritis. Kidney Int (1992) 42(4):903–14. doi: 10.1038/ki.1992.367 1280699

[106] Yung S Cheung KF Zhang Q Chan TM. Mediators of inflammation and their effect on resident renal cells: Implications in lupus nephritis. Clin Dev Immunol (2013) 2013:317682. doi: 10.1155/2013/317682 24171032 PMC3793320

[107] Zhang L Zhang X. Downregulated mir-203 attenuates il-β, il-6, and tnf-α activation in Traf6-treated human renal mesangial and tubular epithelial cells. Int J Clin Exp Pathol (2020) 13(2):324–31. PMC7061798 32211116

[108] Zhang LH Xiao B Zhong M Li Q Chen JY Huang JR. Lncrna Neat1 accelerates renal mesangial cell injury *Via* modulating the mir-146b/Traf6/Nf-Kappab axis in lupus nephritis. Cell Tissue Res (2020) 382(3):627–38. doi: 10.1007/s00441-020-03248-z 32710276

[109] Wu L Han X Jiang X Ding H Qi C Yin Z. Downregulation of renal hsa-Mir-127-3p contributes to the overactivation of type I interferon signaling pathway in the kidney of lupus nephritis. Front Immunol (2021) 12:747616. doi: 10.3389/fimmu.2021.747616 34745118 PMC8566726

[110] Satoh T Akira S. Toll-like receptor signaling and its inducible proteins. Microbiol Spectr (2016) 4(6). doi: 10.1128/microbiolspec.MCHD-0040-2016 28084212

[111] Jamaly S Rakaee M Abdi R Tsokos GC Fenton KA. Interplay of immune and kidney resident cells in the formation of tertiary lymphoid structures in lupus nephritis. Autoimmun Rev (2021) 20(12):102980. doi: 10.1016/j.autrev.2021.102980 34718163

[112] Sung SJ Fu SM. Interactions among glomerulus infiltrating macrophages and intrinsic cells *Via* cytokines in chronic lupus glomerulonephritis. J Autoimmun (2020) 106:102331. doi: 10.1016/j.jaut.2019.102331 31495649 PMC6930355

[113] Yellin MJ Brett J Baum D Matsushima A Szabolcs M Stern D. Functional interactions of T cells with endothelial cells: The role of Cd40l-Cd40-Mediated signals. J Exp Med (1995) 182(6):1857–64. doi: 10.1084/jem.182.6.1857 PMC2192229 7500031

[114] Ramanujam M Steffgen J Visvanathan S Mohan C Fine JS Putterman C. Phoenix from the flames: Rediscovering the role of the Cd40-Cd40l pathway in systemic lupus erythematosus and lupus nephritis. Autoimmun Rev (2020) 19(11):102668. doi: 10.1016/j.autrev.2020.102668 32942031

[115] Zheng N Wang D Ming H Zhang H Yu X. Baff promotes proliferation of human mesangial cells through interaction with baff-r. BMC Nephrol (2015) 16:72. doi: 10.1186/s12882-015-0064-y 25975951 PMC4432501

[116] Da Z Li L Zhu J Gu Z You B Shan Y. Cxcl13 promotes proliferation of mesangial cells by combination with Cxcr5 in sle. J Immunol Res (2016) 2016:2063985. doi: 10.1155/2016/2063985 27672667 PMC5031877

[117] Feng X Wu C Yang M Liu Q Li H Liu J. Role of Pi3k/Akt signal pathway on proliferation of mesangial cell induced by Hmgb1. Tissue Cell (2016) 48(2):121–5. doi: 10.1016/j.tice.2015.12.007 26822343

[118] Dong J Wang QX Zhou CY Ma XF Zhang YC. Activation of the Stat1 signalling pathway in lupus nephritis in Mrl/Lpr mice. Lupus (2007) 16(2):101–9. doi: 10.1177/0961203306075383 17402366

[119] Qing X Pitashny M Thomas DB Barrat FJ Hogarth MP Putterman C. Pathogenic anti-DNA antibodies modulate gene expression in mesangial cells: Involvement of Hmgb1 in anti-DNA antibody-induced renal injury. Immunol Lett (2008) 121(1):61–73. doi: 10.1016/j.imlet.2008.08.007 18822317 PMC2677706

[120] Feng X Hao J Liu Q Yang L Lv X Zhang Y. Hmgb1 mediates ifn-Gamma-Induced cell proliferation in mmc cells through regulation of cyclin D1/Cdk4/P16 pathway. J Cell Biochem (2012) 113(6):2009–19. doi: 10.1002/jcb.24071 22275109

[121] Feng XJ Liu SX Wu C Kang PP Liu QJ Hao J. The Pten/Pi3k/Akt signaling pathway mediates Hmgb1-induced cell proliferation by regulating the nf-Kappab/Cyclin D1 pathway in mouse mesangial cells. Am J Physiol Cell Physiol (2014) 306(12):C1119–28. doi: 10.1152/ajpcell.00385.2013 24760979

[122] Horii Y Iwano M Hirata E Shiiki M Fujii Y Dohi K. Role of interleukin-6 in the progression of mesangial proliferative glomerulonephritis. Kidney Int Supplement (1993) 39:S71–5. 8468929

[123] Kiberd BA Young ID. Modulation of glomerular structure and function in murine lupus nephritis by methylprednisolone and cyclophosphamide. J Lab Clin Med (1994) 124(4):496–506. 7930875

[124] Zhao X Hao J Duan H Rong Z Li F. Phosphoinositide 3-Kinase/Protein kinase B/Periostin mediated platelet-derived growth factor-induced cell proliferation and extracellular matrix production in lupus nephritis. Exp Biol Med (Maywood) (2017) 242(2):160–8. doi: 10.1177/1535370216668050 PMC5167113 27590500

[125] Sun F Teng J Yu P Li W Chang J Xu H. Involvement of tweak and the nf-κb signaling pathway in lupus nephritis. Exp Ther Med (2018) 15(3):2611–9. doi: 10.3892/etm.2018.5711 PMC5795405 29456665

[126] Ligtenberg G Arends S Stegeman CA de Leeuw K. Predictors of renal flares and long-term renal outcome in patients with lupus nephritis: Results from daily clinical practice. Clin Exp Rheumatol (2022) 40(1):33–8. doi: 10.55563/clinexprheumatol/c58c39 33822705

[127] Lech M Anders HJ. The pathogenesis of lupus nephritis. J Am Soc Nephrol (2013) 24(9):1357–66. doi: 10.1681/ASN.2013010026 PMC3752952 23929771

[128] Ma Y Fang L Zhang R Zhao P Li Y Li R. Cyclophosphamide attenuates fibrosis in lupus nephritis by regulating mesangial cell cycle progression. Dis Markers (2021) 2021:3803601. doi: 10.1155/2021/3803601 34820026 PMC8608492

[129] Sepe V Libetta C Giuliano MG Adamo G Dal Canton A. Mycophenolate mofetil in primary glomerulopathies. Kidney Int (2008) 73(2):154–62. doi: 10.1038/sj.ki.5002653 17989649

[130] Gao Y Yang H Wang Y Tian J Li R Zhou X. Evaluation of the inhibitory effect of tacrolimus combined with mycophenolate mofetil on mesangial cell proliferation based on the cell cycle. Int J Mol Med (2020) 46(4):1582–92. doi: 10.3892/ijmm.2020.4696 PMC7447332 32945359

[131] Tani C Elefante E Martin-Cascón M Belhocine M Lavilla Olleros C Vagelli R. Tacrolimus in non-Asian patients with sle: A real-life experience from three European centres. Lupus Sci Med (2018) 5(1):e000274. doi: 10.1136/lupus-2018-000274 30538815 PMC6257376

[132] Tardy M Dold M Engel RR Leucht S. Trifluoperazine versus low-potency first-generation antipsychotic drugs for schizophrenia. Cochrane Database Syst Rev (2014) 7):Cd009396. doi: 10.1002/14651858.CD009396.pub2 PMC11227318 25003310

[133] Wang B Luo Y Zhou X Li R. Trifluoperazine induces apoptosis through the upregulation of Bax/Bcl2 and downregulated phosphorylation of akt in mesangial cells and improves renal function in lupus nephritis mice. Int J Mol Med (2018) 41(6):3278–86. doi: 10.3892/ijmm.2018.3562 PMC5881739 29568865

[134] Wang B Zhou X Wang Y Li R. Trifluoperazine inhibits mesangial cell proliferation by arresting cell cycle-dependent mechanisms. Med Sci Monit (2017) 23:3461–9. doi: 10.12659/msm.902522 PMC5525635 28713151

[135] Aizawa T Imaizumi T Tsuruga K Watanabe S Chiba Y Matsumiya T. Mizoribine selectively attenuates monocyte chemoattractant protein-1 production in cultured human glomerular mesangial cell: A possible benefit of its use in the treatment of lupus nephritis. Nephrol (Carlton) (2014) 19(1):47–52. doi: 10.1111/nep.12171 24134561

[136] Wang H Lu M Zhai S Wu K Peng L Yang J. Alw peptide ameliorates lupus nephritis in Mrl/Lpr mice. Arthritis Res Ther (2019) 21(1):261. doi: 10.1186/s13075-019-2038-0 31791413 PMC6889545

[137] Li Y Yao J Han C Yang J Chaudhry MT Wang S. Quercetin, inflammation and immunity. Nutrients (2016) 8(3):167. doi: 10.3390/nu8030167 26999194 PMC4808895

[138] Liu Y Yu C Ji K Wang X Li X Xie H. Quercetin reduces tnf-Alpha-Induced mesangial cell proliferation and inhibits Ptx3 production: Involvement of nf-kappab signaling pathway. Phytother Res (2019) 33(9):2401–8. doi: 10.1002/ptr.6430 31317585

